# Internal Persistence and External Support—What Makes Chinese Teachers of the Mathematically Gifted Stick to Their Post?

**DOI:** 10.3389/fpsyg.2022.833372

**Published:** 2022-06-20

**Authors:** Lv Sunzhong, Zhang Yong, Lei Peiyao, Xiong Bin

**Affiliations:** ^1^School of Mathematical Sciences, East China Normal University, Shanghai, China; ^2^Shanghai Key Laboratory of Pure Mathematics and Mathematical Practice, Shanghai, China; ^3^School of Mathematics, Yunnan Normal University, Kunming, China

**Keywords:** Mathematical Olympiad, teachers of the mathematically gifted, Herzberg’s two-factor theory, motivation factors, hygiene factors, retention

## Abstract

Mathematically gifted students are precious human resources, educators of which make a great difference in helping them realize their potential. The retention of qualified teachers of mathematically gifted students is an issue worthy of in-depth exploration. In China, mathematics competitions are an important part of mathematics gifted education, and the teaching of the Mathematical Olympiad is a challenging profession with a high attrition rate. This qualitative study takes four seasoned and outstanding Chinese teachers as cases, collects data through individual semi-structured interviews, and uses the thematic analysis method based on Herzberg’s two-factor theory to analyze why they can persist in the field of Mathematical Olympiad teaching for more than 20 years. The results show that the motivation factors affecting retention are recognition, achievement, possibility of growth, work itself, and responsibility, and the hygiene factors are policy and interpersonal relationships. Motivation factors are the main reason for their long-term persistence, while hygiene factors are conducive to their persistence as a whole. Finally, enlightenment to educational policy and school management and suggestions for retention and development of teachers of the mathematically gifted are provided.

## Introduction

Mathematics is the foundation of scientific and technological innovation. The identification and cultivation of mathematically gifted students are not only conducive to releasing potential abilities but also makes contributions to the prosperity of the country. Research on mathematical giftedness is gradually attracting the attention of scholars in different fields, and is still under development (Brody and Stanley, 2005; Phillipson and Callingham, 2009; Myers et al., 2017; Smedsrud, 2018; Leikin, 2021). In practice, diverse activities are prepared and held for mathematically gifted students. As an important part of mathematics gifted education, mathematics competitions play a key role in identifying and cultivating teenagers’ mathematical talents (Kenderov, 2006). Countries around the world hold various primary and middle school mathematics competitions each year, among which the most influential is the International Mathematical Olympiad (IMO).

In China, the Mathematical Olympiad is the training mode for mathematically gifted students with the longest history, the richest educational accumulation, the largest number of participants, and the most obvious Chinese characteristics. China has made brilliant achievements since it first participated in the IMO in 1985. The Chinese national team won first place 22 times and 168 gold medals in total until 2021 (He et al., 2022). The following is a brief introduction to the selection process of the Chinese national team for the IMO. The National Senior High School Mathematics Competition (NSHSMC) is an influential and mass extracurricular activity. About 50,000 Chinese students participate in it every year (Xiong and Jiang, 2021) with about 400 contestants qualifying for the Chinese Mathematical Olympiad (CMO). More than 10 teachers in each province are awarded National Excellent Teacher by the Chinese Mathematical Society because of their students’ outstanding performance. The difficulty of CMO is equivalent to that of IMO; 60 students with the most outstanding performance in CMO will form a National Training Team. These 60 students can be recommended to any top university in China. Six students were selected through several examinations to form the Chinese national team to participate in the IMO of that year (Xiong and Jiang, 2021).

Contestants of IMO and other similar mathematics competitions are top mathematically gifted students who have passed a series of intensive selections. Scholars have carried out relevant research on this special gifted group (Campbell and Walberg, 2010), but empirical studies in this field are few (Jung and Lee, 2021). Teachers play a vital role in improving gifted students’ inventions and creative achievements (Renzulli, 1968; Croft, 2003). Teachers can positively influence gifted students when they are aware of their unique educational needs (Porath, 2009). Gifted students’ success is linked with both specialized education and teachers’ support (Campbell et al., 2000; De and Gubbins, 2011). To provide an adequate educational environment for gifted students, teachers need to be equipped with knowledge about how to educate the talented, including identifying, nurturing, and becoming aware of their social value (Wilhelm and Prado, 2000; Davis et al., 2014). A survey of Chinese Mathematical Olympiad contestants shows that 68% of them believe that the person who has the most influence on the development of their mathematical talent is their math teacher (Zha et al., 1996).

However, only a few studies have focused on teachers of the mathematically gifted (Karp, 2010; Leikin, 2011; Karsenty, 2014). Teacher retention—that is preventing qualified teachers from leaving their positions (Kelchtermans, 2017)—is a topic of concern in the international academic field. If teachers stay in the same position long enough to become more experienced and effective, it will have a positive impact on their work. Teachers of the mathematically gifted need to spend enormous time helping gifted students tap their math potential and meet their special needs. This kind of work will be more complex and cumbersome than general education, while gifted students have different cognitive, emotional, physical, intuitive, and social characteristics compared to their peers (Colangelo and Davis, 1997). Studies have shown that high-quality teachers are likelier to leave their positions (Strunk and Robinson, 2006). Therefore, how to retain them is a topic that education administrative departments and educators should consider. In China, teachers of the mathematically gifted should not only build the math foundation for all students, but also be responsible for the Mathematical Olympiad for mathematically gifted students. This profession is demanding and requires teachers’ selfless dedication. Usually, many teachers will choose to leave this post after working for some time or will only be responsible for routine math teaching. Teaching the mathematically gifted is a challenging profession with a high attrition rate. Therefore, it is of great significance to study the influencing factors of teachers engaged in Mathematical Olympiad teaching and explore the reasons for their long-term persistence in front-line teaching.

## Literature Review

### Conceptions of Giftedness and the Characteristics of Gifted Students

Diverse and competing conceptualizations of giftedness exist (Sternberg and Davidson, 2005), for example, emphasizing multiple categories, types, and components of giftedness or intelligence vs. highlighting a basic factor. Interdisciplinary exploration is undertaken to better understand giftedness. Despite all the differences in points of view, there are some commonalities in these diverse viewpoints. Sternberg and Ambrose (2021) summarized 10 uniform points of agreement in diverse viewpoints on giftedness and talent, such as motivation, passion, and attitudes are important to giftedness. The characteristics of gifted children are tied to how they are identified for special programming. As a summary overview, Davis et al. (2014) presented a collection of recurrent positive and negative characteristics of gifted students.

Mathematical giftedness is sometimes seen as a specific part or kind of giftedness. Given the domain-specificity of mathematical giftedness, it always implies a collection of certain mathematical abilities (such as mathematical sensibility, exceptional memory, rapid content mastery and structuring, atypical problem solution, and preference for abstraction, interest, and enjoyment of mathematics) and personal qualities (such as intellectual curiosity, willingness of exertion, and joy and interest in problem solving; Singer et al., 2016).

### Programs for Mathematically Gifted Students

Davis et al. (2014) summarized programs to help meet the educational needs of gifted and talented children, which involve those as simple or as complex as 10 categories, such as part-time acceleration to a higher grade for one or two subjects, grade-skipping, and special schools for the gifted. In general, programs or activities for mathematically gifted students consist of in-school programs (such as ability grouping, self-contained classes, specialized schools, acceleration, and skipping) and extra-curricular programs (such as recreational mathematics and competitions; Singer et al., 2016). Despite the variety of programs available, there is a lack of systematic empirical studies on various programs to gain a better understanding of their suitability for the realization of students’ mathematical potential.

As a basic type of extracurricular program, mathematics competitions and related activities are an important or the only way to identify, challenge, and enhance gifted students’ mathematical expertise. Tracking mathematics contestants and investigating their views on mathematics competition experiences may help to evaluate the effectiveness of mathematics competitions on the development of contestants’ mathematics abilities as well as future career choices (Campbell et al., 2000; Taylor, 2017). A systematic review of existing surveys concerning Mathematical Olympiad winners showed that most achieved strong development and held positive attitudes toward their mathematics competition experiences (He et al., 2022).

### Study of Teachers of Mathematically Gifted Students

The value of teachers in education appears to be especially salient in nurturing gifted students (Renzulli, 1968; Croft, 2003). Croft (2003) noted that gifted students are more profoundly impacted by their teachers’ attitudes and actions than are other students. However, relatively few empirical studies have focused on the understanding of teachers of gifted learners in comparison to those of gifted learners (Chan, 2011).

Existing studies mainly focus on teachers’ conceptions of giftedness, competence characteristics, and professional development. Based on a systematic review of related studies, Whitlock and DuCette (1989) believe that the outstanding characteristics of teachers of the gifted are their enthusiasm and self-confidence, strong achievement orientation, and recognition as outstanding educators. Feldhusen (1997) summarized the characteristics and competencies of successful teachers of gifted and talented students, which include being highly intelligent, achievement oriented, skills in teaching thinking skills, problem solving, and so on. Chamberlin and Chamberlin (2010) summarized the abilities of teachers as the knowledge needed by gifted students, promoting high-level thinking and problem solving, creativity, developing diversified courses, and creating a safe, flexible, and student-oriented environment. VanTassel-Baska (2005) found that the ideal profile of a teacher for the gifted included competencies, such as mastery of disciplinary content knowledge, positive interaction with students, and use of diverse teaching strategies. Park and Steve Oliver (2009) found that science teachers developed content-specific teaching strategies based on their understanding of gifted students. Studies have also revealed that many teachers believe that gifted students tend to show less social adjustment than average-ability students (Baudson and Preckel, 2016).

Specifically, studies of teachers of mathematically gifted students are scarce (Singer et al., 2016). In an earlier study, Stanley (1975) mentioned the abilities that these teachers must possess in grades 4–12. Karp (2010) interviewed two Russian teachers, studied their teaching characteristics, and discussed the role of mathematically gifted students in the teachers’ self-development. Leikin (2011) analyzed the teacher’s teaching characteristics and summarized the qualities that teachers need when cultivating mathematically gifted students, such as embracing challenges, a great sense of humor and creativity, a passion for mathematics, etc. Shayshon et al. (2014) compared mathematics teachers’ perception of gifted students in three countries. The research showed that these teachers think they can cultivate mathematically gifted students, and that different mathematics learning backgrounds and class teaching experiences of different sizes will affect their perception ability. Karsenty (2014) stressed that teachers of the mathematically gifted in middle schools should have abundant mathematical knowledge, teaching skills, and personal social quality. Hoth et al. (2016) revealed the relationship between teachers’ professional knowledge and their skills to identify and support mathematically gifted students. It was found that teachers who have difficulties in logical reasoning and understanding mathematics structures also have difficulties identifying mathematically gifted students.

The above studies were based in Western cultures, and studies that focused on the characteristics and competencies of Chinese teachers of mathematically gifted students are limited (Chan, 2001, 2011; Cheung and Hui, 2011). Empirical studies focusing on Chinese teachers who teach the Mathematical Olympiad have not yet been found.

### Teachers’ Retention and Job Satisfaction

Retention of an adequate supply of competent teachers is critical to provide a high-quality education to every student (Guarino et al., 2006; Borman and Dowling, 2008). The following parts in this section mainly discuss Herzberg’s two-factor theory, which offers a useful lens to determine factors motivating or demotivating teachers to continue or quit teaching and some representational studies of teacher retention.

Two-factor theory of Herzberg et al. (1959) focuses on motivation factors and hygiene factors (see Table 1). Motivation factors can provide work satisfaction, and hygiene factors can be used to eliminate dissatisfaction (Herzberg et al., 1959; Herzberg, 1966). The core of the two-factor model is the relationship and difference between motivation and hygiene factors. These two aspects can be combined to discuss the influencing factors of the work. The two-factor theory has been widely used to study the motivation of different organizations, including the application of this model in educational research, such as the retention and attrition of teachers in ordinary K–12 public schools (Adrianzen, 2012; Wood, 2014) and rural areas (Winfield, 2019). At the same time, this theory has been used by some scholars to analyze the influencing factors of teachers’ professional development (McMillan et al., 2016).

**Table 1:** Summary of job attitude factors by Herzberg (1966) and Herzberg (2008).

Motivation Factors	Hygiene Factors
1. Recognition	1. Salary
2. Achievement	2. Interpersonal relations
3. Possibility of growth	3. Supervision–technical
4. Advancement	4. Company policy/administration
5. Responsibility	5. Working conditions
6. Work itself	6. Personal life
	7. Status
	8. Job security

It is found that hygiene factors influencing teacher retention mainly include relationship with school leaders and colleagues, work pressure, salary, etc. Firstly, school leaders influence teachers’ work and students’ learning (Grissom, 2011; Shi et al., 2020). Learning-oriented leadership has a positive impact on teachers’ professional development (Hallinger and Liu, 2016; Liu et al., 2016). Secondly, relationships with colleagues will influence teachers’ professional development, work fatigue, and sense of job belonging (Waddell, 2010; Kelchtermans, 2017). Work pressure will also affect teachers’ work, which comes from students’ examinations, school management policies, and workload (Towers and Maguire, 2017; Barni et al., 2019). Finally, teachers’ salary affects their professional decision-making (Borman and Dowling, 2008).

Motivation factors influencing teacher retention mainly include professional development, teachers’ autonomous decision-making, self-efficacy, sense of responsibility, teachers’ enthusiasm for education, etc. First, professional development affects teachers’ retention (Guarino et al., 2006), and the ability of students has an impact on teachers’ professional development (Matteucci et al., 2017; Edinger and Edinger, 2018), which indirectly influences teacher retention. Secondly, teachers’ autonomous decision-making, self-efficacy, and sense of responsibility can affect their job satisfaction, thus influencing their retention (Matteucci et al., 2017; Edinger and Edinger, 2018). There is a certain connection between teachers’ sense of responsibility and self-efficacy, and it also affects their work motivation (Lauermann and Karabenick, 2011; Lauermann, 2014). Finally, teachers’ enthusiasm for education also affects their job retention (Towers and Maguire, 2017), students’ love for mathematics (Russo et al., 2021), and their own teaching quality (Russo et al., 2020).

### The Present Study

Teachers need to implement an educational strategy that can be most appropriate for gifted students to reach their potential. To meet their special learning needs, teachers need long-term persistence to improve their teaching. Therefore, it is noteworthy to analyze the factors influencing their retention. In China, teaching mathematically gifted students is challenging, so these teachers have a high attrition rate. Based on Herzberg’s two-factor theory, this study explores the influence of motivation and hygiene factors on teachers’ retention. This theory provides an intuitive perspective for analyzing four Chinese teachers of the mathematically gifted who have been engaged in teaching the Mathematical Olympiad for more than 20 years so as to further analyze the motivation and hygiene factors affecting their persistence in this field. The application of the theoretical framework commenced to formulate the following research questions:

1. What extrinsic (hygiene) factors influence participants’ willingness for retention?
2. What intrinsic (motivators) factors influence participants’ willingness for retention?
3. How do these factors influence participants’ willingness for retention?

## Methodology

This study is based on a qualitative approach, with semi-structured interviews as the core method of data collection. A case-study approach was adopted to explore the storied experiences of four outstanding teachers. The greatest strength of a case study is its ability to exemplify larger processes or situations in a very accessible, concrete, immediate, and personal manner (Duff, 2012). These case studies will help capture the complexity of their experiences and offer an in-depth understanding of the issue derived from a real-life setting (Yin, 2017). Hence, the qualitative approach is suitable to obtain rich and valid information on factors influencing the four teachers’ retention and job satisfaction. Similarly, semi-structured interviews in the qualitative approach were acknowledged as an appropriate means for analyzing the complex issue of motivation and were favored over quantitative information, which cannot, by itself, afford comprehensive accounts of the motivational practices and challenges in teaching the Mathematical Olympiad.

Herzberg’s theory, as an effective concept, is highly recognized and broadly used in research globally. The theory shows that when employees are provided with challenging yet enjoyable work that offers them great achievements, they are motivated (Dartey-Baah and Amoako, 2011). This study aims to use Herzberg’s theory to explore what motivates teachers who teach mathematically gifted students in China to remain in their position and how these factors impact their job satisfaction regardless of a huge workload. Furthermore, only through the qualitative method of semi-structured interviews can we grasp the motivation behind teachers’ behavior.

### Participants

Four cases were recruited from a wider mixed research study carried out to investigate teachers’ source of knowledge of the Mathematical Olympiad. In the survey, we collected 108 valid questionnaires, of which only three were from female teachers. This is also in accordance with the conditions in China, where most teachers who teach the Mathematical Olympiad are male. A purposive sampling approach was taken to recruit the participants who were included in the sample based on their judgment of their typicality or possession of the particular characteristics being sought (Cohen et al., 2011). The teachers in the sample had to fulfil two criteria: 20 or more years of teaching and excellent achievement in Mathematical Olympiad teaching. Finally, four outstanding Chinese teachers of the mathematically gifted were recruited, who were males from four different provinces in China. They have rich experience in cultivating mathematically gifted students and are excellent representatives of professional teachers. Table 2 shows the basic information of the four cases, which includes pseudonyms.

**Table 2:** Basic information of participants.

Pseudonyms	Age	Gender	Mathematical Olympiad Teaching Years	Degree	Academic Title
Wang Chun	50	Male	29	Master	Senior
Li Dong	46	Male	20	Bachelor	Sub-senior
Zhang Xia	53	Male	30	Bachelor	Sub-senior
Zhao Qiu	57	Male	26	Bachelor	Sub-senior

In China, senior teacher is the highest academic title for teachers in middle school, and a sub-senior teacher is second to a senior teacher (Tan, 2013). Except for Li Dong, the teachers are special-class teachers, which are an honorary title for a very small group of teachers who have many years of teaching experience and are recognized by the education department as people who have made outstanding contributions to the teaching practice (Tan, 2013). In terms of Mathematical Olympiad teaching honors, the four cases have won National Excellent Teacher, and Zhang Xia and Zhao Qiu are also Mathematics Olympiad senior teachers (Zhou, 1990). In terms of work experience, Wang Chun initially taught normal students Mathematical Olympiad courses as a university teacher, and later applied to transfer to the local high school. Li Dong is the head of the school’s math teaching and research group. Like Zhao Qiu, he had been working in middle school and had changed his work unit once during that time. Zhang Xia has been working in the same school without changing his work unit. In terms of students’ achievement, five of Wang Chun’s students won medals in CMO, and 20 of them won the first prize in NSHSMC. Li Dong helped a student win the IMO gold medal, and 11 students won first prize at NSHSMC. Zhang Xia trained more than 50 participants to win medals in the CMO, of which five students won gold medals. Zhao Qiu cultivated three IMO gold medal participants, and 33 students won CMO gold medals.

### Data Procedure

The research was conducted in accordance with the ethical standards and checked by the Committee on Human Research Protection of East China Normal University. After ethical approval, individual semi-structured interviews were utilized as a major data source, supplemented with relevant documentation and online communication to achieve data triangulation. The main contents of the interview are as follows: (1) when did you decide to engage in Mathematical Olympiad teaching, and why did you choose to do so?; (2) Please talk about your daily work as a teacher for the mathematically gifted, and what abilities or qualities do you need?; (3) In your teaching career, what are the important factors that affect your willingness to engage in teaching the Mathematical Olympiad?; and (4) Can you tell us reasons as you know why math teachers chose or gave up Mathematical Olympiad teaching? Probing questions were also asked to gather more detailed information or to clarify some points. Each participant completed in-depth interviews lasting about 60–90 min *via* the Internet at their convenience, and the interviews were recorded and transcribed verbatim with their consent. Any names or specific locations mentioned during the interviews were anonymized.

### Data Analysis

Thematic analysis offers an accessible and theoretically flexible approach to analyzing qualitative data (Braun and Clarke, 2006). So, inductive thematic analysis was employed in this study. The data analysis procedure consisted of three stages. First, each researcher read and re-read each transcript thoroughly to become familiar with the data. The second stage involved generating initial codes. In line with the research questions, two researchers independently coded each case by NVivo12.0 and then compared the codes. Whenever there was disagreement, the two researchers discussed to identify the source of disparity and to reconcile the differences. Third, the data of the four cases were cross-analyzed to check the similarity and then combined. Similar codes were clustered under broader and more abstract categories based on Herzberg’s two-factor theory. All categories were grouped under two themes, namely motivator factors and hygienic factors. Each category and theme was discussed and reviewed by the researchers. Finally, the synopses of the themes, categories, and codes that were attained from the data are shown in Table 3.

**Table 3:** Summary of categories and specific code.

Motivation factors	Specific code
Recognition	Students’ trust, leadership’s support, parents’ support, and social evaluation
Achievement	Solve problems, students’ achievement, students’ growth and success, teaching honor, and meaningful work
Possibility of growth	Think about questions, solve problems, communicate with students, and classroom teaching
Work itself	Interested in mathematics, love teaching, like solving mathematical problems, like communicating with excellent students
Responsibility	Work overtime at night and during holidays, think about mathematical questions in spare time, and purchase books at their own expense
Hygiene factors	Specific code
School’s policy	Policy guarantee, reward system, the system of faculty team, and priority of professional title evaluation
Interpersonal relationship	Leadership’s attention, colleagues’ cooperation

## Findings

The findings are presented in three parts, namely the analysis and summary of the motivation factors and hygiene factors affecting the four cases.

### Motivation Factors

The motivation factors affecting teachers’ long-term engagement in Mathematical Olympiad teaching mainly include recognition, achievement, possibility of growth, work itself, and responsibility. On the one hand, the work of cultivating mathematically gifted students can be widely recognized, which will bring a sense of achievement to teachers, and this process is also accompanied by teachers’ own growth, which will affect the acquisition of recognition and achievement. On the other hand, the work itself has its own professional characteristics, and this job has special requirements for responsibility. The work itself and the responsibility are more like the basic attributes of the job and teaching career. Therefore, the first three closely related factors are taken as one category, and the last two factors are classified as one category.

#### Recognition, Achievement, and Possibility of Growth

##### Recognition

External recognition is an important driving force for the four cases to remain in their work. Recognition is divided into both within and outside the working environment. The former mainly includes recognition for their teaching from school leaders, colleagues, and students’ parents and gratitude for their hard work. The latter mainly includes praise for their teaching achievements from society.

Both Wang Chun and Zhang Xia mentioned recognition from schools, parents, and students. Wang Chun particularly emphasized that social affirmation and students’ gratitude are important driving forces for his work. Zhang Xia’s special emphasis is placed on students’ recognition. He felt that students’ recognition of his teaching is a kind of trust in him, which promotes his work.

Wang Chun: If you can teach Mathematical Olympiad Courses, people will respect you. Though the job is hard, it is never short of labor because it is proof of competence, which matters to the teacher.

Zhang Xia: The leader empowers you to take this job, which is a signal of trust and appreciation. Students choose you as they think they’ll learn a lot from you, which is also a kind of trust. I have a passion for teaching. For example, 1 day after class, my students said to me, “You gave very good lectures and I learned a lot from you.” Hearing this, I felt that I had his approbation.

Regarding recognition, Li Dong and Zhao Qiu stated relatively little, but they all believed that it would promote their work. Li Dong believed that parents’ recognition is conducive to the smooth development of teaching activities. Zhao Qiu held that good talents will be recognized by students and colleagues, and students’ achievements are the main reason for being recognized by others. Zhao Qiu also considered that, to be recognized by students, one must first have good teaching ability in routine courses.

Zhao Qiu: When your students get good scores, people hold a positive attitude toward you. However, if they do poorly, people may not hold a negative attitude toward you, but suspicion emerges, at least, and your flame of passion in teaching may be put out as a result.

##### Achievement

While being recognized by the outside world, teachers will also get a sense of achievement. Also, the achievements brought about by the cultivation of gifted students also include students’ growth and success, their achievements, and the study of math problems. All cases mentioned that students’ growth and success will make teachers feel successful in their work and give a strong sense of achievement. Among them, three teachers mentioned their students’ relevant experiences in postgraduate and doctoral studies and were proud of them. For example, Wang Chun said that it is a wonderful thing to “gather together the best talents of the world and cultivate them,” and Zhao Qiu shared many small stories about his students.

Li Dong: On the whole, students with experience in the Mathematical Olympiad had great expectations. As I remember, most of them received a doctoral degree later. As students in 2017, only 15 of them continued on Mathematical Olympiad learning. They received a bachelor’s degree and directly enrolled in a Ph.D. program.

Zhao Qiu: This student is talented, not only in the Mathematical Olympiad, but also in other areas. After graduation, she went to Berkeley to study statistics. After obtaining her doctoral degree, she went to Harvard to do her postdoctoral studies. The 2021 Nobel Prize in Economics has just been announced. One of its laureates is a cooperator of my student. Part of the mathematical derivation in economics was carried out by her.

Except for Zhang Xia, the other three teachers mentioned the students’ math scores. They believed that helping students get good grades was not only a direct driving force, but also the main way to obtain a sense of achievement.

Wang Chun: I felt a sense of achievement when my students received various awards, which are rewards to me and bring a sense of achievement.

Meanwhile, both Wang Chun and Zhang Xia think that successfully solving a Mathematical Olympiad problem can also bring significant happiness, and they have the experience of summarizing ideas and publishing papers in the process of Mathematical Olympiad teaching, producing a sense of achievement.

Zhang Xia: Before this internship, I had been thinking about the inverse question of a question, and it turned out to be a functional equation. And I made a systematic study on it then. Later, I refined the content, turned it into a contesting question, and published it in High-School Mathematics.

##### Possibility of Growth

In terms of the possibility of growth, the teachers’ ideas were basically the same. They all believed teaching benefits teachers as well as students (this is an old saying in *Record on the Subject of Education*), which means that in the process of teaching, students and teachers promote each other and grow together and emphasized two aspects: their own thinking on mathematical problems and communication with students.

On the one hand, they often must spend considerable time thinking about mathematical problems and figuring out how to teach them. This process improved their mathematical and teaching levels. On the other hand, gifted students have strong mathematical thinking and many ideas about problems. Communication with them helps to improve teachers’ professional level. All of these promote self-growth.

Li Dong: Once you meet a good student, as a teacher, you are lucky. You may also learn along with them. They are young and have many fresh ideas beyond the imagination of a teacher. In the exchanges of ideas with students, the teacher also learns a lot, and it’s a kind of progress to the teacher as well.

Zhang Xia: Of course, it’s helpful. Exchanges with the students play an important role in the development of a teacher. I would still like to talk with my students. I had more exchanges with my high school students for the Mathematical Olympiad. Frequently, I discuss a certain math question with them because their thoughts are the most direct.

Zhao Qiu: It’s a pleasure to be with excellent students. Teaching is a way of learning. We also learned a lot from our students and improved our skills.

#### Work Itself and Teacher’s Responsibility

##### Work Itself

Genuine interest in the field of mathematically gifted education emerged as a significant factor that motivated teachers to stay in the present study. The four cases in this study love mathematics and teaching. On the one hand, the experience in students’ arena made them become interested in studying mathematical problems, so they felt that this job was full of challenge and joy. On the other hand, they like teaching gifted students. They thought this job could provide them with opportunities to communicate with excellent students, and it is meaningful to cultivate people because it is conducive to discovering and training students with mathematical talents.

Three cases participated in the Mathematical Olympiad when they were in primary and secondary school. One case did not, but he also showed a strong interest in mathematics when he was young. Wang Chun participated in math contests in primary and secondary schools and won awards. Li Dong said that he taught himself a little knowledge about Mathematical Olympiad and participated in math contests, but he did not win a prize. Zhang Xia recalled that he liked to delve into higher mathematics problems when he was an undergraduate. He felt that Mathematical Olympiad was relatively low-end scientific research. Zhao Qiu showed mathematical talent in high school. Although he had not received Mathematical Olympiad training, he also participated in math contests. When he was an undergraduate, he was very interested in elementary number theory, which was very helpful to his current work.

Wang Chun: When I was in primary school, I participated in math contests and won awards, at the county and regional level. The experience of these competitions and awards has had a positive impact on my enthusiasm for mathematics and my current teaching of the Mathematical Olympiad.

Zhang Xia: I think the Mathematical Olympiad can teach students ways of thinking. Actually, solving Mathematical Olympiad problems is also a process of scientific research. I deem it basic scientific research. However, this task is prepared by others, and you know there is a clear answer ahead. The outcome of ordinary scientific research is unknown.

Zhao Qiu: I took elementary number theory as the optional course at the undergraduate stage. This course played a great role in my later work. At that time, I did not know what kind of work I would be engaged in later. It was entirely out of interest.

It is worth mentioning that although three teachers except Zhang Xia had the experience of changing work units, they always adhered to the front-line teaching of the Mathematical Olympiad. Wang Chun is a typical representative. He used to work at a university and was invited to give lectures on the Mathematical Olympiad in middle school. In this process, he fell in love with communicating with excellent students, so he applied to be transferred to a prestigious local middle school for Mathematical Olympiad teaching. Li Dong applied to transfer to the school because the principal of the school he currently works for attached great importance to him, and he also hopes to make a breakthrough at work. Zhao Qiu did not give much explanation about his job transfer.

Wang Chun: At that time, I was teaching in a university. I also went to junior or senior high schools to assist teachers in guiding mathematics. In this process, I felt that I’d like to make exchanges with outstanding students, and I found out that I love teaching Mathematical Olympiad. Therefore, I decided to apply for a job transfer and worked in a senior high school in 1998.

Li Dong: Our principal told me clearly that, “if you work here, I can offer you good conditions so that you can focus on Mathematical Olympiad teaching.” At that time, I had accumulated some materials and wanted to make a breakthrough in the Mathematical Olympiad. So I came to this school. After I came, school leaders paid great attention to me, which was an encouragement to me, so I said to myself I cannot fail them.

##### Responsibility

Responsibility is also an important factor for four teachers’ teaching career. Teachers are regarded as a sacred profession in China and need to be responsible to students. Due to the particularity of experienced and excellent teachers’ work, they are more bound to students. The four cases were dedicated and responsible. They all mentioned their extra pay, such as working overtime during winter and summer vacations and weekends, accompanying students in the evening, studying Mathematical Olympiad topics in their spare time to maintain their professional level, etc. They have spent a lot of time, and they do not ask for anything in return, which is also the teacher’s mentality in Chinese traditional culture.

Li Dong: Actually, we barely have weekends, vacations, or holidays. By and large, we seldom had rest days during summer vacation, and we normally stayed with our students in school. We usually hold Mathematical Olympiad trainings on weekday nights or Saturdays, so some students live at school on Saturdays, and we need to visit them.

Zhang Xia: Our only idea is to teach our students. We have made many contributions. Everybody rests on Saturdays, but we must work because the trainings must be arranged on weekends. We even work on legal holidays. Our students also made more effort, so we took on this job. We had only one or two teachers who taught Mathematical Olympiad, and we worked without complaint, not for money or fame. At that time, this was our inner thinking.

Both Wang Chun and Zhang Xia mentioned online training during the pandemic. Li Dong regularly communicated with students to relieve their pressure. Also, books are mentioned by Li Dong and Zhao Qiu, as they have purchased many at their own expense for Mathematical Olympiad training.

Zhao Qiu: I have been buying all I can get on the internet since 2007, including all exercise books and materials about Mathematical Olympiad. I have been doing this for 3 or 4 years. I bought them on kongfz.com. I spent about hours on this website every weekend searching for various old exercise books about Mathematical Olympiad. It cost me about 30,000 RMB.

### Hygiene Factors

The hygiene factors affecting teachers’ long-term participation in teaching Mathematical Olympiad include policy and interpersonal relationships, personal life, salary, and working conditions. The four cases mentioned the first two factors more frequently, and the latter factors were mentioned less relevantly.

#### Policy and Interpersonal Relationships

##### School’s Policy

School policy is the main influencing factor. It is an important prerequisite for teachers to stick to Mathematical Olympiad teaching and make themselves work more firmly. Wang Chun and Zhao Qiu mentioned that the school will give some material rewards and spiritual encouragement if its students get good grades. Moreover, both believed that teachers of the mathematically gifted had more opportunities, which may be awards or preferential treatment in the evaluation of professional titles.

Wang Chun: If students made certain achievements in the competition, the school would give them material and spiritual awards.

Zhao Qiu: Our school invested heavily in math contests. A gold medal at IMO would bring you a lot of money from the school, which will take effect this year. In the past, our school also had awards for students who achieved good scores in math contests. Generally, the school holds a positive attitude toward it.

Li Dong felt that the school had a good policy guarantee for his work, including the priority of professional title evaluation and the formation of a Mathematical Olympiad team. With the support of the education department, the school where Zhang Xia worked established a Mathematical Olympiad class recruited from the whole province. Now, although such classes have been canceled, he continued to teach Mathematical Olympiad.

Li Dong: School policies provides guarantee for us, including paying fees for training. There is a system of simple rules in our school that is carried out by teachers of every grade. The school leader often organized meetings for us, offering an opportunity for exchanges. The whole team is harmonious, in my view.

Zhang Xia: Now, our school policies have had a few changes. A math teacher from an experimental class helped teach the content of the college entrance examination. Therefore, I can focus on teaching plane geometry, number theory, and in-equation and combinatorial mathematics.

Finally, Wang Chun also mentioned that the school has certain restrictions on the teaching of the Mathematical Olympiad. For example, students cannot leave homework of other subjects behind during Mathematical Olympiad learning. However, he understands and agrees with this because he thinks it is also for the better development of students.

Wang Chun: The school takes some specific measures. The school leaders feel that students cannot gamble to engage in the Mathematical Olympiad and let students fall behind in other subjects. Therefore, for Mathematical Olympiad courses, there are regulations on how much time can be spent a week, what level of students can stop classes to learn math, and how long to suspend classes at most.

##### Interpersonal Relationship

Colleagues and leadership are two important factors in interpersonal relationships. Except for Wang Chun, the other three cases believed that leaders attach great importance to their work, which is a guarantee of their work quality. At the same time, Li Dong and Zhao Qiu felt that their colleagues were also very cooperative with their work and occasionally brought them some help, such as helping him manage the students. These harmonious interpersonal relationships provide them with support in many aspects and improve their work efficiency.

Li Dong: In a word, I think school leaders support this activity. And I get along well with the colleagues around me. Sometimes, when I went out, our students came to the class on time, and they discussed math questions, and then I may ask my colleague to help them.

Zhao Qiu: Our head teacher and grade dean are young, and we have a good relationship in private. I received their strong support. When I was discussing some questions with them, they had a positive attitude. Our cooperation is delightful.

Wang Chun believed that before NSHSMC, he often occupied the time of his colleagues. However, they support him because their common goal is to better cultivate students.

Wang Chun: Especially before the math contests–there are about eight students–the two-week class may have to be stopped. Then colleagues may feel that I take up their time and they worry about whether these students’ other homework will fall behind, but every teacher’s objective is for the better growth of students.

#### Personal life, Salary, and Working Conditions

##### Personal Life

Although busy work may occupy the personal lives of four cases, they thought it did not negatively impact their personal lives. On the contrary, one teacher thought that this way of work could inspire his family. Wang Chun believed that this encourage his son and created a positive role model for him. Under his influence, his son also majored in mathematics during his undergraduate studies. Zhao Qiu thought that his family generally supported him, but he did not give a specific explanation. Both Li Dong and Zhang Xia held that work does not have much impact on life; accordingly, life factors will not affect work.

Wang Chun: The most important thing is that my family can see my efforts and thinking, which has a positive effect on them, especially my child. He knows that I am thinking. Sometimes, I feel that it plays a good role in guiding him.

##### Salary

Salaries seem to have little impact on them. Wang Chun did not mention salary much. Li Dong simply mentioned that there would be some class fees in Mathematical Olympiad teaching, but did not speak in detail. Zhang Xia thought his salary was not very high, but he did not think it was particularly important. Zhao Qiu just mentioned the school’s reward system for competitions but did not mention salary.

Zhang Xia: Our training is not for money though it’s a hard work. At that time, my salary was very low. I remember it was five or so RMB per class hour, and no bonus.

##### Working Conditions

In terms of working conditions, the four cases did not describe too much except for the teaching materials. They evaluated some books that showed that teaching materials are vital. Wang Chun thought good materials should be original, and it is best to have a difficulty coefficient for each topic. Li Dong argued that as a quality book, the topic types should be summarized in place, and it is better to combine the author’s own problem-solving experience. Zhang Xia said that most of the books he used had only the solution process, but he could not find the entire thought process. Zhao Qiu said that good books should be more systematic, with a more thorough explanation. He also counted some books he had read in those years.

Li Dong: *Study of Mathematical Olympiad* is really a good one, for its clear clarification of solving questions. Of course, the author has already solved these questions, and wrote down his experience in this book. Therefore, this book is a quality one for the use of education, such as *Classics of Mathematical Olympiad Problems* published by Hunan Normal University, which clearly sorts out the types of questions.

### Summary of Influencing Factors

To make the data more specific and intuitive, this study created a word frequency analysis of the interview texts using NVivo12.0, and counted the keywords related to the topic based on the two-factor model. Due to space limitations, Table 4 shows only the frequency of some keywords. Combined with word frequency analysis, this study summarizes the findings as follows.

**Table 4:** Keywords and frequency of each motivation factor.

Work Itself		Achievement, Recognition, Possibility of Growth		Responsibility	
Keywords	Frequency	Keywords	Frequency	Keywords	Frequency
Students	451	Score	65	Willing	30
Mathematics	133	Affirmation	49	Help	19
Teaching	116	Ability	48	Investment	14
Problems	90	Solve	27	Effort	9
Time	75	Examination	25	Persistence	8
Study	59	Support	21	Extra	5
Thinking	35	Improve	20		
Communication	31	Recognition	17		
Parents	31	First prize	15		
Love	25	Ideas	15		
Number theory	21	Cultivate	14		
Research	19	Growth	14		
Materials	17	Peking University	14		
Difficulty	17	Training team	12		
Combination	15	Win the prize	12		
Solve problems	14	Gold medal	11		
Interests	10	National team	10		
Weekends	9	Doctor	10		
Plane geometry	9	Master	8		
Difficulty	9	Answer	8		

#### Motivation Factors

As to “work itself,” Table 4 shows that the three keywords “student,” “mathematics,” and “teaching” appear most frequently. They are closely related to the work of teachers of the mathematically gifted; “students” is the main service object, “teaching” is the main task, and “mathematics” is the main teaching content. Words related to teaching behavior, such as “learning,” “thinking,” and “communication,” “research,” “solve problems,” “discussion,” “interaction,” “analysis,” and “expansion,” are descriptions of some main behaviors that may occur in or out of class. As the main content of teaching, the word “mathematics” also appears frequently. The words related to “mathematics” mainly include “problems,” “number theory,” “combination,” “plane geometry,” “algebra,” and so on. These high-frequency words show the working scenario of four teachers. Among them, solving mathematical problems is daily work, and the content of problems involves number theory, combination, and plane geometry. According to the interviews, due to the content of the Mathematical Olympiad, its teaching is difficult and comprehensive, and it will take up much of teachers’ holidays and rest time, as the table includes “time,” “weekend,” “difficulty,” and other words. Wang Chun said that he even thinks about mathematics problems in his dreams.

In addition to the above three high-frequency words outlining the working scenario, there are also other high-frequency words, such as “love,” “interests,” “enthusiasm,” which show four cases’ positive attitudes toward the work. The enthusiasm for mathematics and the preference for gifted students are the important reasons why the four cases have been working for more than 20 years. Although Li Dong and Zhang Xia are involved in Mathematical Olympiad teaching because of the arrangement of the school, and Zhang Xia has no experience in participating in relevant competitions, the four cases have one thing in common—that is, they show their interest and love in mathematics when they were students, like to challenge mathematical problems. From mathematically gifted students to teachers of the mathematically gifted, those teachers who participated in math contests as a student may continue to pursue the dream of teaching, and place this dream on their students, which should be a main reason why they work on Mathematical Olympiad teaching. In their work, these teachers will encounter many gifted students with special needs education and will face many seemingly complex mathematics problems. However, they gradually started to enjoy exchanging ideas with these students. From them, these teachers seem to find the shadow of themselves, which makes them feel gratified. At the same time, in the process of thinking, they will also find these mathematical problems fascinating and challenging so that they can feel joy from the work itself. All four teachers liked to discuss math problems with excellent students. Li Dong said it was like doing simple scientific research. Gifted students and Olympiad problems have become important partners in their lives, increasing their interests and enthusiasm in their work.

In terms of “recognition,” “achievement,” and “possibility of growth,” the most frequent words were “score,” “affirmation,” and “solve.” The first is the recognition of teachers, which is mainly related to “achievement” and “affirmation.” The words related to “achievement” are “examination,” “first prize,” “winter camp,” “win the prize,” “training team,” “gold medal,” and “national team.” The words “affirmation,” “support,” and “recognition” in Table 3 mainly refer to the recognition obtained by teachers. Recognition is partly and directly linked to students’ performance. Especially from parents and school leaders, good results can gain more recognition. Although recognition will be related to material gains, it has a direct influence on students’ learning and teachers’ teaching. Parents’ recognition is the catalyst for students’ learning, because it can provide students with better learning conditions, while parents who do not recognize students’ work rarely offer help, and even interfere with their Mathematical Olympiad learning. These parents may think that only the college entrance examination is important. The recognition of leaders is conducive to the continuation of Mathematical Olympiad teaching and the smooth development of Olympiad activities because leaders directly manage teaching. Li Dong mentioned that the leaders of his original unit did not pay attention to the Mathematical Olympiad, so he changed jobs to a school where the leaders attached great importance to it. Also, there is recognition from students. Considering four cases, students’ recognition of teachers is more reflected in teaching quality and mathematical level. This recognition may be a kind of trust, admiration, and emotional sustenance. When students graduate, they also express their appreciation to their teachers. Currently, recognition is more like a kind of gratitude.

The second is “achievement,” which is related to “score,” “affirmation,” and “solve.” Students’ scores bring the most direct sense of achievement to teachers, and the honor brought by excellent students’ achievements, such as National Excellent Teacher, is an official affirmation and will indirectly bring teachers a sense of achievement. Because students’ achievements are the most direct result of teachers’ work, these honors are awarded to teachers as a reward and feedback for their work. Also, the success of students is also an indirect affirmation for teachers. The words “Peking University,” “doctor,” and “master” were frequently mentioned by teachers who showed their pride and satisfaction brought by students’ success. For example, they felt that their work was meaningful, and they have a sense of pride in cultivating talents for the country. There is also a word related to achievement, “solve,” which relates to studying mathematical problems. For example, Wang Chun receives a sense of satisfaction when solving mathematical problems successfully. He also published an article on solving mathematical problems. He thought this process was very meaningful and therefore gained a sense of achievement.

The last one is “possibility of growth.” As mentioned above, the training of gifted students also promotes the development of teachers. Whether it is the thinking of mathematical problems, the design of teaching process, or the communication with students, teachers need to study deeply, which is also accompanied by the improvement of teachers’ own ability. In terms of growth, words such as “ability,” “improvement,” “growth,” and “development” occur. All cases mentioned that communication with mathematically gifted students enabled them to grow better because their mathematics level was not necessarily higher than that of their students. In front of students, teachers have multiple identities, sometimes teachers, and sometimes students, because they can learn from students’ homework and test papers, listen to their ideas in class, and discuss mathematical problems with them after class.

For the four cases, a sense of responsibility is an important guarantee of staying at their posts. The words in the column of “responsibility” include “willing,” “help,” “investment,” “effort,” “persistence,” and “extra.” These all indicate the unique sense of responsibility of the four cases, which is embodied in their unrepentant help and wholehearted investment in students. In school, students must participate in many subjects. Mathematical Olympiad is only a supplement to mathematically gifted students. Teachers can only use their spare time to teach. These teaching hours are basically arranged for non-working hours, such as weekday evenings, weekends, and winter and summer vacation. In additions to these hours, they are also responsible for routine college entrance examination teaching during their daily working hours. Therefore, their workloads are heavier, and their working hours are longer than those of ordinary teachers. At the same time, compared with conventional teaching and lesson preparation, Mathematical Olympiad teaching is much more complex. Teachers need to spend more time collecting data and thinking about problems. Therefore, in addition to significant classroom teaching time, teachers will also spend a lot of time preparing lessons. Furthermore, due to the great mental pressure experienced by some students, teachers should communicate with them regularly to understand their inner thoughts. Much of this extra work, in addition to a small class fee in teaching, is unpaid and only supported by the teachers’ sense of responsibility. Facing the consumption of time and energy, teachers also bear great psychological pressure. One teacher said that his anxiety and pressure could only be faced alone and relieved by time. Another teacher said that he was always worried that his math level would decline and would take the initiative to solve math problems as soon as he was free. Another teacher mentioned that as he gets older, he sometimes doubts his education level and teaching ability, which makes him anxious. In fact, these anxieties also reflect their strong sense of responsibility and mission—that is, being responsible for every student.

#### Hygiene Factors

Table 5 summarizes the words of three dimensions related to hygiene factors. The words related to the keyword “personal life” include “children,” “family” and “home.” As mentioned, the cultivation of mathematically gifted students had little impact on the personal life of the four cases. The words in school policy include “team,” “opportunity,” “award,” and “bonus.” Awards and bonuses are more of an opportunity, and team construction is vital for sustainable school development. There are main hygiene factors affecting teachers’ engagement in Mathematical Olympiad teaching. First, the four cases pointed out that the class fees for Mathematical Olympiad teaching and conventional teaching is basically the same, while the content of Mathematical Olympiad teaching is much more complex and takes more time. To encourage their work, the school sets awards and bonuses directly linked to students’ competition results. In addition, Li Dong’s school has relatively united a team for teaching the Mathematical Olympiad. The regular communication and mutual assistance of team members are conducive to the formation of a series of shared resources, to reduce the workload of teachers, and to promoting the construction of a school-based curriculum. Finally, Wang Chun mentioned that their school has strict requirements on students’ time to learn Mathematical Olympiad, and this time is directly linked to students’ comprehensive performance, because if students spend too much time on Mathematical Olympiad learning, it may affect their comprehensive performance. To better engage in Mathematical Olympiad teaching, Li Dong changed his work unit once, because the original unit competition system was not very sound. Good hygiene factors can reduce the impact of negative factors on work and let teachers work at ease.

**Table 5:** Keywords and frequency of each dimension of hygiene factors.

Personal Life		School’s Policy		Interpersonal Relationship	
Keywords	Frequency	Keywords	Frequency	Keywords	Frequency
Children	8	Team	16	Leaders	23
Family	5	Opportunity	10	Principal	8
Home	5	Award	6	Colleagues	7
		Bonus	6	Head teacher	5

In terms of “interpersonal relationship,” frequently mentioned keywords were “leaders,” “principal,” “colleagues,” and “head teacher.” “Leaders” appeared frequently among which “principal” was the most important, because they can directly affect teaching. Leaders can formulate the management system of the teaching plan, which impacts teachers’ daily work. In addition to mathematics learning, gifted students should also participate in class activities and other courses, so the relationship between teachers of the mathematically gifted and other teachers is vital. From the interview data, the support of colleagues can reduce the workloads of teachers of the mathematically gifted. For example, the head teacher can help them obtain basic student information and provide psychological guidance to students. Other teachers can also occasionally help supervise students’ self-study. These harmonious relationships are conducive to the cohesion of the learning atmosphere.

## Discussion

This study investigated the teaching careers of four outstanding Chinese teachers of the mathematically gifted and explored the impact of various specific factors on their teaching of the Mathematical Olympiad from two aspects: motivation factors and hygiene factors.

In China, the Mathematical Olympiad is an important part of mathematically gifted education (Xiong and Jiang, 2021). Mathematical Olympiad teaching has high requirements for teachers’ personal ability and working hours. Also, in many schools, teachers of the mathematically gifted undertake conventional courses (Zha et al., 1996). A common phenomenon is that many teachers leave this post after several years and are responsible only for routine teaching. The four cases in this study belong to a very small number of special cases that have remained in Mathematical Olympiad teaching for many years. The in-depth investigation of these factors can provide insights into the long-term retention and professional development of teachers of the mathematically gifted.

Through the thematic analysis based on the two-factor theory, this study found that teachers’ persistence in Mathematical Olympiad teaching is mainly driven by motivation factors, such as recognition, achievement, enthusiasm for the work itself, and their sense of responsibility. Also, hygiene factors will also support or hinder their willingness to work, mainly including school policy, management, and interpersonal relationships. Working conditions and personal life have little impact on their work, probably because their schools are some of the best local schools with sound working conditions, high social status, and their families understand and respect their work. According to the two-factor theory, hygiene factors are less important than motivation factors. Motivation factors directly affect job satisfaction, and the regulation of hygiene factors can intervene in dissatisfaction (Herzberg et al., 1959; Herzberg, 1966). The lack of both may lead to brain drain.

According to the research results, almost all the factors affecting the work of the four cases were related to the teachers themselves, students, and school colleagues. They are the most key figures affecting teachers’ work and learning (Kelchtermans, 2017). Next, combined with the Herzberg’s two-factor theory, this study discusses the main factors affecting teachers’ work from three aspects: the teachers themselves, students, and interpersonal relationships.

The teachers themselves mainly involve motivation factors, such as the work itself, achievement, the possibility of growth, and responsibility. First, the enthusiasm for mathematics was the common feature of the four cases, which was not only the original cause of their choice for Mathematical Olympiad teaching, but also the important reason why they remained in this post for many years. Teachers’ positive attitudes toward math, such as their confidence in the subject, are closely related to their enthusiasm for math teaching (Russo et al., 2020). Excellent mathematics teachers have a strong interest in math and challenging problems (Karp, 2010; Leikin, 2011). Their love for the subject can also improve students’ interest in learning. At the same time, it will also stimulate teachers to continuously improve teaching quality, to reduce teachers’ job tiredness and termination (Frenzel et al., 2016), which is also consistent with this study. Secondly, teachers’ own thinking and solving of mathematical problems can inspire students’ sense of achievement. It is one of the unique “personal attributes” possessed by excellent teachers (Chan, 2001). It can sustain teachers’ love for their work and encourage them to work more enthusiastically. At the same time, the four cases in this study have a strong sense of responsibility. They sacrifice significant spare time to think about math problems and accompany students at school. This working spirit will encourage their students to study harder and obtain better results. This sense of responsibility is also one of the most important reasons for them to stick to their work. Previous studies have found that teachers’ sense of responsibility is important because they will work actively and efficiently even without external control and supervision (Lauermann and Karabenick, 2011; Lauermann, 2014). This sense of responsibility will also promote teachers’ self-growth (Lauermann and Karabenick, 2011).

Mathematically gifted students are an important driving force to support the four excellent teachers in remaining in their careers. This is consistent with the existing research findings; that is, students with high cognitive levels and excellent academic performance contribute to the retention of teachers (Guarino et al., 2006; Borman and Dowling, 2008). The factors related to mathematically gifted students are mainly motivation factors, such as recognition, achievement, the possibility of growth, and the work itself. Recognition mainly comes from students and their parents. Students’ recognition of teachers is more inclined toward ability and affection, while parents’ recognition is more inclined toward students’ achievements. In addition to recognition, teachers can gain a sense of achievement given by students. One is the sense of achievement from students’ excellent results. However, taking students’ results as the standard for evaluating teachers’ work will lead to teachers’ frustration and self-doubt due to their pursuit of high standards of teaching achievements, leading to their attrition (Kelchtermans, 2017; Towers and Maguire, 2017). The other comes from the growth and success of students. Students’ achievements will enable teachers to gain teaching honor and recognition from others, and students’ development will make them aware of the significance of this job, which leads them to be more motivated in this job. Finally, mathematically gifted students will drive the growth of the teachers themselves (Leikin, 2011). In the process of cultivating gifted students, teachers’ views on gifted students will change positively, and their abilities will be improved to meet the learning and emotional needs of gifted students (Cheung and Hui, 2011). In addition to problem-solving ability, when students express a deeper understanding of mathematical concepts, their teachers’ ability will be further stimulated and their happiness will be further enriched (Russo and Russo, 2019; Russo et al., 2021).

The school has an important impact on teachers’ work willingness and professional development, and colleagues, including leaders and other teachers, are the key figures affecting this. The recognition and trust of colleagues will affect teachers’ job satisfaction (Waddell, 2010; Kelchtermans, 2017). School leadership is a key factor (Hallinger and Liu, 2016; Liu et al., 2016). Leader recognition will influence teachers’ working attitude, and then their self-learning. Also, leaders participate in the formulation of school systems and policies. Efficient leadership builds a highly professional environment full of respect, care, trust, and understanding. In a campus with a sound atmosphere, leaders will understand, support, and appreciate teachers; thus, teachers will feel that they are also influential in the school, which is conducive to retention. On the contrary, a lack of leadership support is the key reason for teachers’ leaving (Gallant and Riley, 2017). The above findings are supported by the investigation of the four cases in this study. One teacher changed to another school because the former leaders did not support the Mathematical Olympiad. Also, the cooperation of colleagues has also brought great work convenience to the four cases. Research shows that when teachers have more efficient colleagues, students’ academic performance will improve significantly (Jackson and Bruegmann, 2009). Building a Mathematical Olympiad team is a way of in-school cooperation for teachers of the mathematically gifted in this study. Members of the team will communicate regularly and shoulder their responsibilities to each other, which is a strong reason for the four cases remaining committed to their work. Shared responsibility is an important factor in building a high-quality team (Simmons and Magiera, 2007), which includes teaching, evaluation, and other tasks. Cooperation between them can improve teachers’ professional development ability and communication ability (Friend, 2008; Brinkmann and Twiford 2012).

## Conclusion and Implications

This study explores the reasons behind four outstanding Chinese teachers’ persistence in Mathematical Olympiad teaching for more than 20 years. Considering the youth and high mobility of teachers of the mathematically gifted, why can they persist in this special field for so long? The results show that both motivation and hygiene factors affect teachers’ choice to stay in the post of training mathematically gifted students. Recognition, achievement, possibility of growth, the work itself, and responsibility are the main motivation factors affecting teacher retention, and the most important hygiene factors are interpersonal relationships, management, and policy. At the same time, hygiene factors are also the main source of teachers’ negative emotions. Schools can enhance teachers’ job satisfaction by adjusting motivation factors and controlling the impact of hygiene factors on teachers, so as to reduce their dissatisfaction with their work, which is conducive to their retention. This study also confirms that the Herzberg’s two-factor theory can be used as an effective tool to analyze teachers’ job satisfaction retention. It also has two important implications for the Ministry of Education and school management, especially in cultivating and managing teachers of the mathematically gifted.

The Ministry of Education should pay attention to the professional development of teachers who teach the Mathematical Olympiad. On the one hand, the education department can organize mathematicians and mathematics educators to carry out training programs on nurturing mathematically gifted students; on the other hand, it should focus on construction of teachers’ learning resources, and provide professional, classic, readable, and instructive books in line with teachers’ demands and teaching practices.

School leaders should create a comfortable working environment for teachers who teach the Mathematical Olympiad. First, leaders should support and respect them, fully consider their contributions, and give recourse support when making school policies. Second, school leaders should actively build a team of teachers for Mathematical Olympiad to promote communication and cooperation among them. Thirdly, school leaders should strengthen the publicity of the educational achievements of talented students, such as tracking their sustainable development, and publicizing the outstanding work of students and teachers. Finally, school leaders should not only evaluate teachers based on their students’ achievement tests, but also apply scientific and reasonable evaluation standards to ensure and improve the quality of teachers.

This study contributed to providing certain information about a special group of Chinese teachers of the mathematically gifted, which is unexplored, and scholars do not know well enough. The analysis of four teachers’ experiences and perceptions pertinent to working could help gain insights into what underlies teachers’ decisions to stay in the mathematically gifted education field and what could be done to support teacher retention in the field.

## Limitations and Suggestions for Further studies

First, the participants were four teachers of the mathematically gifted from four different schools. Due to the small number of samples and the fact that the four schools are famous schools with good working conditions, the generalizability of the conclusion is relatively weak. Under the theory of Herzberg’s two-factor theory, the importance of influencing factors cannot be ranked. Future research can investigate more teachers of the mathematically gifted to portray more concise patterns of important details, continue to explore the influencing factors of their work, and develop relevant scales to rank the importance of these influencing factors. We can further investigate the reason for the attrition of teachers who left this position to gain more insights. Second, when the subjects were interviewed, much of the data involved memories and reflections of past experiences, and the contents may be distorted. Therefore, future studies should try to collect data from multiple information sources. Questionnaire surveys and classroom observations can be combined to obtain more data from teachers. Also, perspectives such as students, colleagues, and school leaders can help to view this special occupation more objectively.

## Data Availability Statement

The raw data supporting the conclusions of this article will be made available by the authors, without undue reservation.

## Ethics Statement

The studies involving human participants were reviewed and approved by University Committee on Human Research Protection of East China Normal University. The patients/participants provided their written informed consent to participate in this study.

## Author Contributions

LS contributed to the conception and design of the study. LS and ZY collected data and drafted sections of the manuscript. LS, LP, and ZY did the data analysis. XB secured funding for the study. LS, LP, and XB rewrote and revised the manuscript. All authors contributed to the article and approved the submitted version.

## Funding

This study was funded by a grant from the Shanghai Key Laboratory of Pure Mathematics and Mathematical Practice 18dz2271000.

## Conflict of Interest

The authors declare that the research was conducted in the absence of any commercial or financial relationships that could be construed as a potential conflict of interest.

## Publisher’s Note

All claims expressed in this article are solely those of the authors and do not necessarily represent those of their affiliated organizations, or those of the publisher, the editors and the reviewers. Any product that may be evaluated in this article, or claim that may be made by its manufacturer, is not guaranteed or endorsed by the publisher.
